# The mystery of abdominal snow-grip sensation: emphysematous cystitis complicated with bladder gangrene and rupture—case report and literature review

**DOI:** 10.3389/fmed.2024.1408646

**Published:** 2024-11-15

**Authors:** Yiwen Zhou, Yuxi Cai, Yi Yang, Chenyang Xu, Jianbin Xiang, Zujun Fang, Shanhua Mao

**Affiliations:** ^1^Department of Urology, Huashan Hospital, Fudan University, Shanghai, China; ^2^Department of Sports Medicine, Huashan Hospital, Fudan University, Shanghai, China; ^3^Department of Gastrointestinal Surgery, Huashan Hospital, Fudan University, Shanghai, China

**Keywords:** urinary tract infection, type 2 diabetes, emphysematous cystitis, gangrenous cystitis, urinary bladder rupture, *Candida*

## Abstract

Emphysematous cystitis (EC) refers to a rare form of complicated urinary tract infection (UTI), which is typically observed in elderly females with severe diabetes mellitus. EC was triggered by bacteria, producing gas filled cysts in the bladder wall and lumen, the most common of which are *Escherichia coli* and *Klebsiella pneumoniae*. Since EC could be potentially life-threatening, severe form of EC (like septic shock) can cause gangrenous cystitis (GC). This case report describes an 85-year-old female patient with type 2 diabetes mellitus (DM) who presented with EC and bladder gangrene due to the growth of *Candida glabrata* in urine. Our case describes patient’s progression in detail, ranging from the initial clinical manifestations to specific management actions, which may provide new insights and ideas for the diagnosis and treatment of such disease.

## Highlights

- Emphysematous cystitis (EC) refers to a rare form of complicated urinary tract infection (UTI), which is typically observed in elderly females with severe diabetes mellitus.
- EC complicated with bladder perforation is quite uncommon in clinical practice and can lead to serious consequences such as sepsis in the absence of timely diagnosis.
- EC is usually triggered by bacteria, the most common of which are Escherichia coli and Klebsiella pneumoniae, but EC caused by fungi, such as *Candida glabrata* in this case, has barely been reported.
- Our case described patient's progression in detail, ranging from the initial clinical manifestations to specific management actions, which may provide new insights and ideas for the diagnosis and treatment of such disease.
- This report, except for case presentations, also reviews a series of case reports on EC and summarizes the possible pathogenesis of EC, which may provide new ideas for the management of this gas-producing microbial infection.

## 1 Introduction

Emphysematous cystitis (EC) refers to a relatively rare and potentially life-threatening disease characterized by gas within the bladder wall and lumen due to gas-forming bacteria, with a reported mortality rate of 7% (1, 2). Hyperglycemia serves as a major risk factor for EC, which is commonly seen in elderly females with diabetes mellitus (DM) and is present in 50–70% of such patients (2). Other risk factors include chronic urethritis, urethral outlet obstruction, indwelling urethral catheters, neurogenic bladder and immunodeficiencies (3). The pathogenesis of this disease is not fully understood. Among the most common causative agents are gas-forming bacteria such as *Escherichia coli, Klebsiella pneumoniae and Enterococci* (4). However, the clinically severe form of EC (e.g., septic shock, acute abdominal pain syndrome) could evoke gangrenous cystitis (GC), constituting a surgical emergency, which has become rare after the advent of antibiotics (5).

We report a case of emphysematous cystitis complicated with gangrene of the bladder in an 85-year-old female who presented with abdominal pain and specific snow-grip sensation. Simultaneously, to the best of our knowledge, this case is the second documented case of *Candida glabrata* as the causative organism following the documentation by Viktoriya et al. (6). Our case may provide new insights and ideas for the diagnosis and treatment of such unique disease.

## 2 Case presentation

An 85-year-old female patient who had been experiencing generalized abdominal pain for 2 days was admitted to the Emergency Department of Huashan Hospital. She was diagnosed with type 2 DM 20 years ago along with unsatisfactory glycemic control. One month ago, she fell down accidentally and suffered pain in her lower limbs, suspected pelvic fracture, and indwelling urinary catheterization ever since. In addition, she exhibited episodes of speech confusion 6 months ago, raising the suspicion of early-stage Alzheimer’s disease (AD). Treatment with Memantine (10 mg qd po) was initiated, and her symptoms have recently exacerbated. Finally, she underwent a uterine tumor resection 50 years ago, which was followed by a total hysterectomy due to postoperative hemorrhage.

On arrival at the Emergency Department: the patient was not fully conscious along with rapid pulse (98 BPM). Blood pressure (BP) temporarily stabilized at 120/70 mmHg, and she had an indwelling urinary catheter with hematuria. Abdominal examination revealed a slightly distended abdomen with positive periumbilical tenderness, mid-lower abdominal tenderness, rebound tenderness and loss of bowel sounds. The patient had negative Murphy’s sign. It was noteworthy that twisting sensation (or snow-grip sensation) could be palpated in her lower abdomen. In addition, digital rectal examination (DRE) indicated no palpable mass, no blood staining of the fingertip.

Emergency laboratory tests showed abnormal blood count (WBC 28.80*10^9^/L, N% 94.0), mildly impaired renal function (Creatinine 120 μmol/L), hyperglycemia (41.7 mmol/L), urine erythrocyte (++++), urine leukocytes in full view, and poor Blood Gas Analysis (Table 1).

**Table 1:** Emergency laboratory examinations.

**Blood routine**	**WBC**	**N%**	**HB**	**PLT**		**CRP**	
	28.80 × 10^9^/L	94.0%	102 g/L	391 × 10^9^/L		121.49 mg/L	
**Urinalysis**	**URO**	**BIL**	**KET**	**RBC**	**WBC**	**PR**	**TPC**
	(−)	(−)	(+)	(++++)	Full view	(++)	174.4/μL
**Coagulation**	**TT**	**PT**	**APTT**		**DDI**	**FIB**	
	17.6 s	12.2 s	30.4 s		3.66FEUmg/L	9.4 g/L	
**Liver Function**	**TAST**		**ALT**	**BIL**		**ALB**	
	21 U/L		21 U/L	7.2 μmol/L		31 g/L	
**Renal Function**	**Cr**		**BUN**		**UA**		
	120 μmol/L		3 mmol/L		0.165 mmol/L		
**Electrolyte**	**K** ^**+**^			**Na** ^**+**^			
	4.5 mmol/L			145 mmol/L			
**ABG**	**pH**	**PO**_**2**_	**SpO**_**2**_	**PCO**_**2**_	**AG**	**BE**	**AB**
	7.30	72.8 mmHg	94.5%	43.2 mmHg	18.5 mmol/L	−4.0 mmol/L	20.0 mmol/L
**AMY**	<30 U/L						
**BG**	41.7 mmol/L						
**BK**	(−)						

WBC, white blood cells; N, neutrophils; HB, hemoglobin; PLT, platelet; CRP, C-reactive protein; URO, urobilinogen; BIL, bilirubin; KET, ketone body; RBC, red blood cells; PR, protein; TPC, total plate count; TT, thrombin time; PT, prothrombin time; APTT, activated partial thromboplastin time; DDI, D-Dimer; FIB, fibrinogen; TAST, total aspartate transaminase; ALT, alanine transaminase; ALB, albumin; Cr, creatinine; BUN, blood urea nitrogen; UA, uric acid; ABG, arterial blood gas; AG, anion gap; BE, base excess; AB, actual bicarbonate; AMY, amylase; BG, blood glucose; BK, blood ketone.

**Figure 1:**
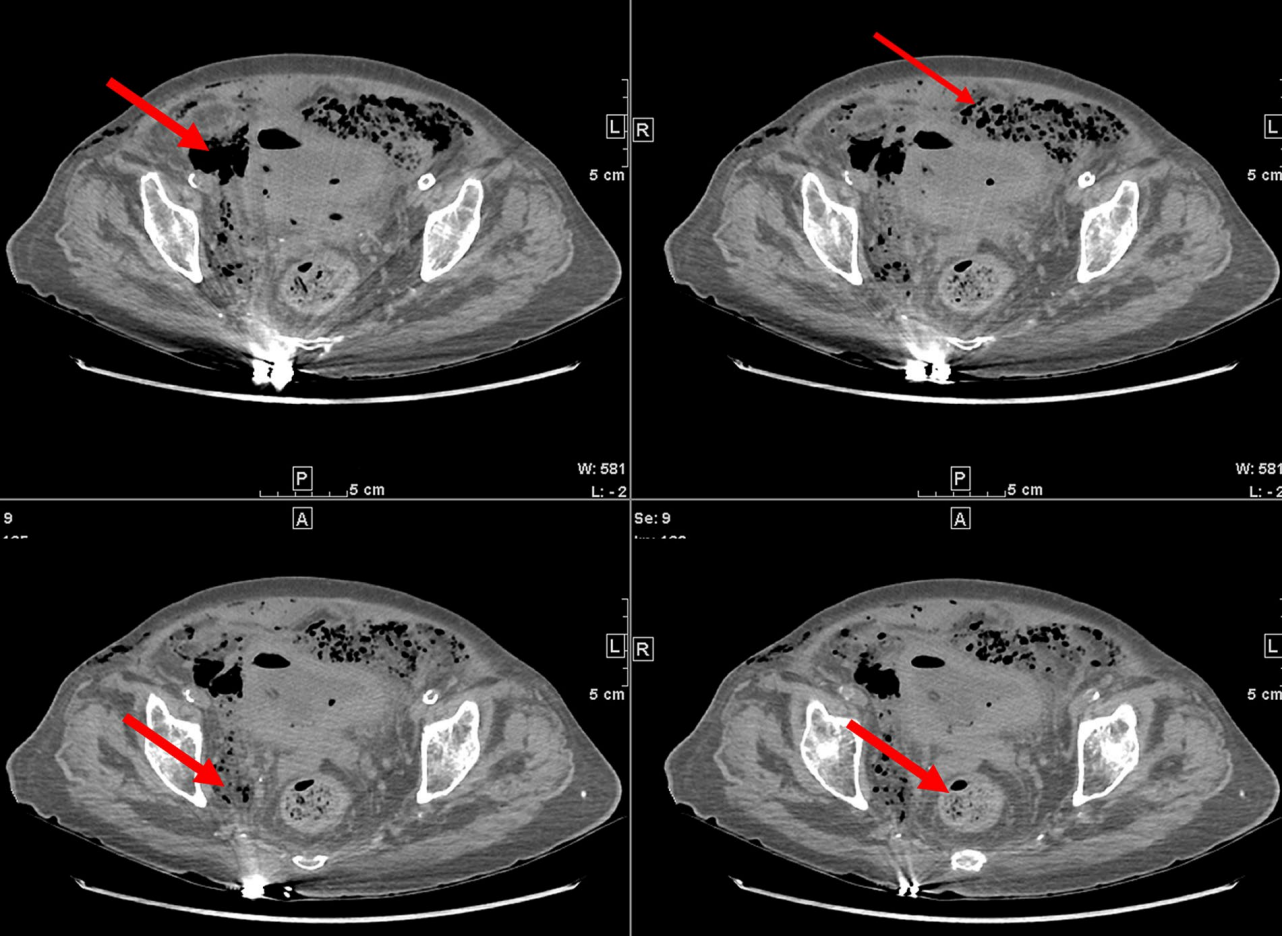
Nonenhanced computed tomography (CT) images showed gas around the bladder (red arrows).

**Figure 2:**
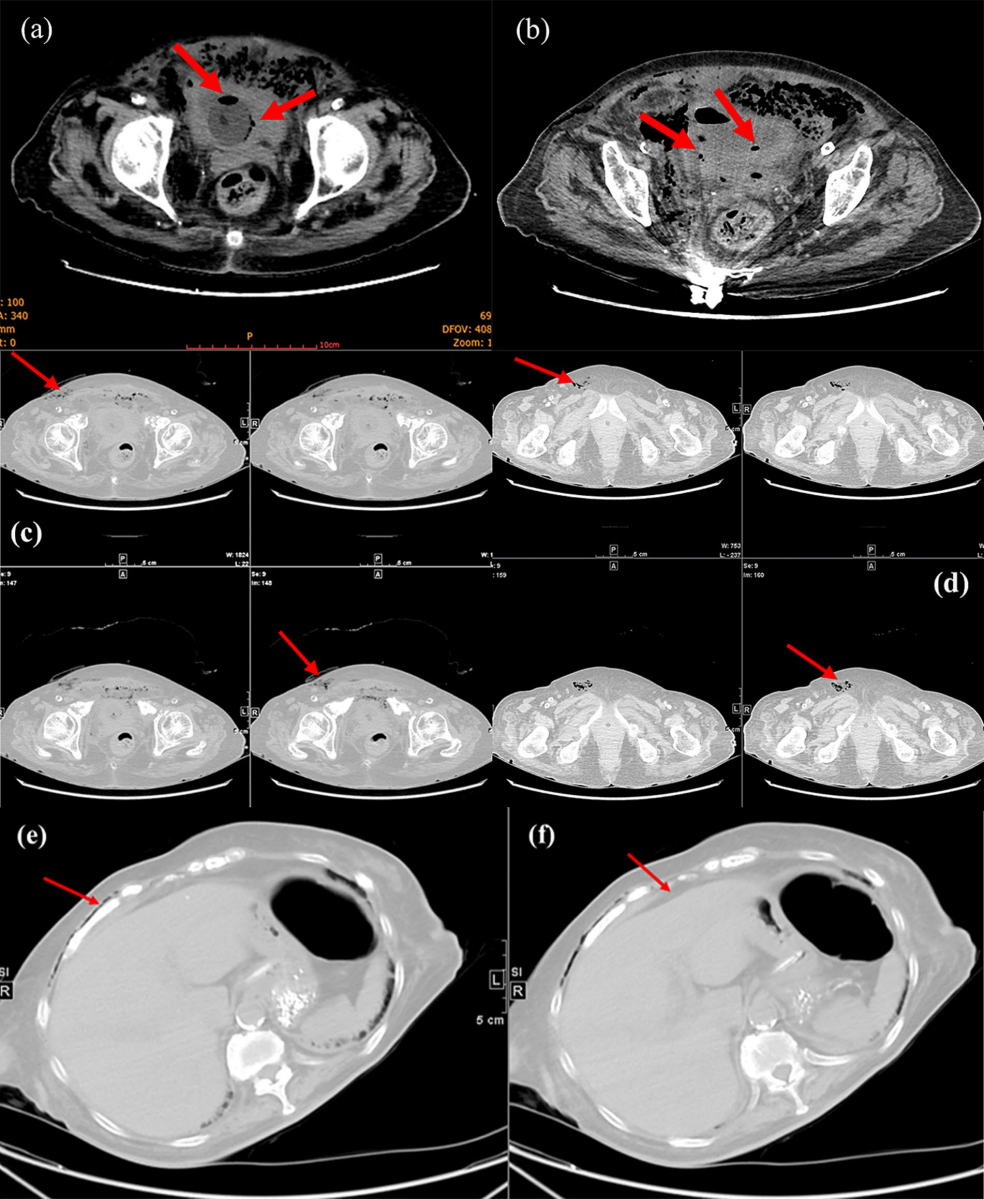
Magnified view of CT demonstrated pneumatization of the bladder wall and bladder cavity (red arrows, **a** and **b**). Lower abdominal CT showed subcutaneous gas in the abdominal wall (red arrows, **c**) and gas in the femoral canal (red arrows, **d**). Upper abdominal CT indicated subcutaneous gas in the chest wall (red arrows, **e**) and no free subdiaphragmatic gas (red arrows, **f**).

The patient then underwent a non-contrast abdominopelvic computed tomography (CT) scan. The abdominopelvic CT not only revealed gas in the bladder wall and bladder cavity, but also hypodense shadows around the bladder and even in the subcutaneous tissues of abdominal and chest wall (Figures 1, 2). Consequently, preliminary radiographic impression was retropneumoperitoneum with possibility of retroperitoneal or interpositional cavity organ perforation.

Initially, the patient was managed with fasting, followed by the implementation of monitoring protocols, including electrocardiographic (ECG) monitoring and BP assessment. Then, she received volume expansion (1,500 mL in 3 h), anti-infective therapy (1.0 g meropenem intravenously), correcting acidosis, and hypoglycemic treatment (endocrinology consultation). Nevertheless, the patient subsequently experienced a progressive drop in BP (127/70 mmHg at 2:00 a.m. → 120/60 mmHg at 4:00 a.m. → 100/50 mmHg at 10:00 a.m. → 89/43 mmHg at 12:00 a.m.), which presented as infectious shock. While we tried out maintained BP with vasopressor drugs, the decision was made by the Multi-Disciplinary Team (MDT) to perform the surgery (Laparoscopic examination under general anesthesia) as soon as possible.

**Figure 3:**
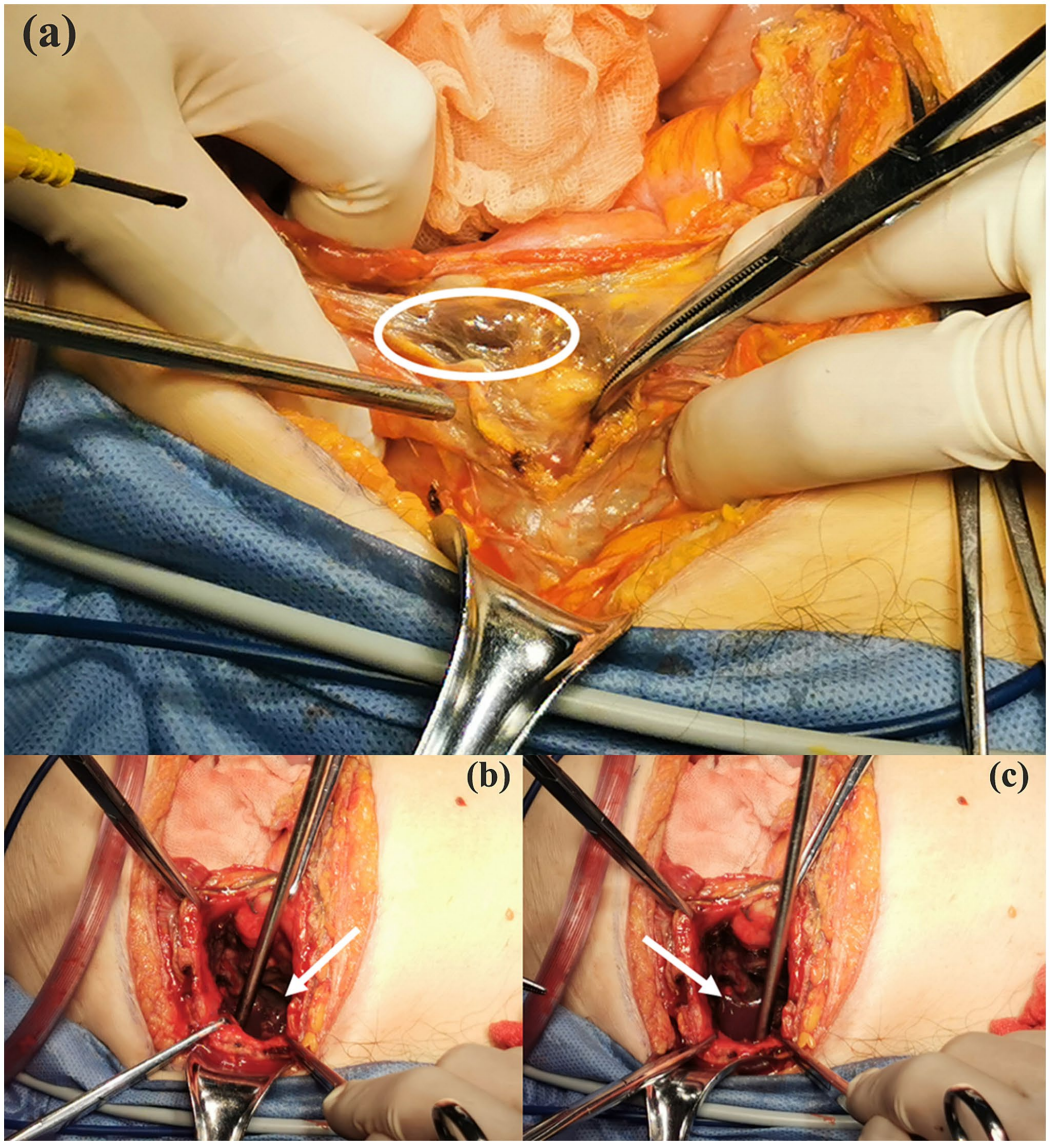
Intraoperative detected gangrenous tissue of bladder mucosa (white circle area, **a**), active hemorrhage and clot filling in the bladder observed during intraoperative exploration (white arrows, **b** and **c**).

During the operation, we separated the small intestine that was adherent to the bladder and discovered black patchy changes in the serosal layer of the bladder. Given the consideration of gangrene of the bladder, we decided to accelerate the surgical approach with open laparotomy. After the cystotomy, extensive gangrene of the bladder vesicle mucosa and localized perforation of the bladder apex were observed (Figure 3a), which was also confirmed by the water injection test. Additionally, we also witnessed active hemorrhage in the bladder wall and blood clots filling the bladder cavity after dissecting the plasma membrane (Figures 3b,c). Then, we performed a partial cystectomy on the area of necrotic tissue in the bladder and gathered the necrotic mucosa for bacterial culture. Immediately after that, we closed the remaining bladder in two layers using 2–0 absorbable stitches and left a cystostomy tube to the skin opening. Finally, after hemostasis of the trauma, we placed a drainage tube in the pelvis as well, which was directed through the right abdominal Trocar hole and secured. After the surgery, the patient was transferred to the Intensive Care Unit (ICU) for anti-infective managements.

**Figure 4:**
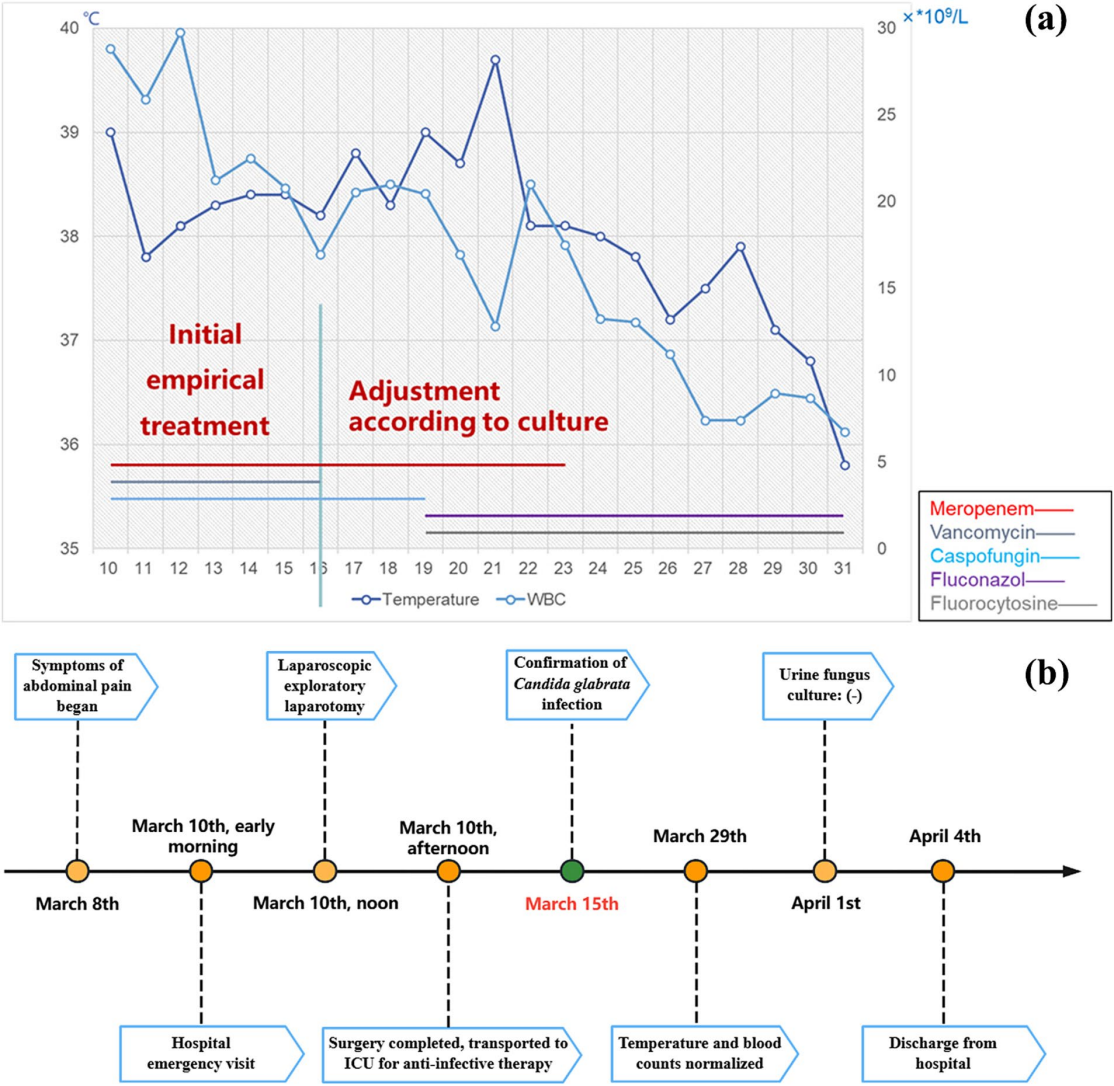
Anti-infective regimen and real-time recordings of patient temperature and white blood cells **(a)**. Timeline of patient hospitalization **(b)**.

**Figure 5:**
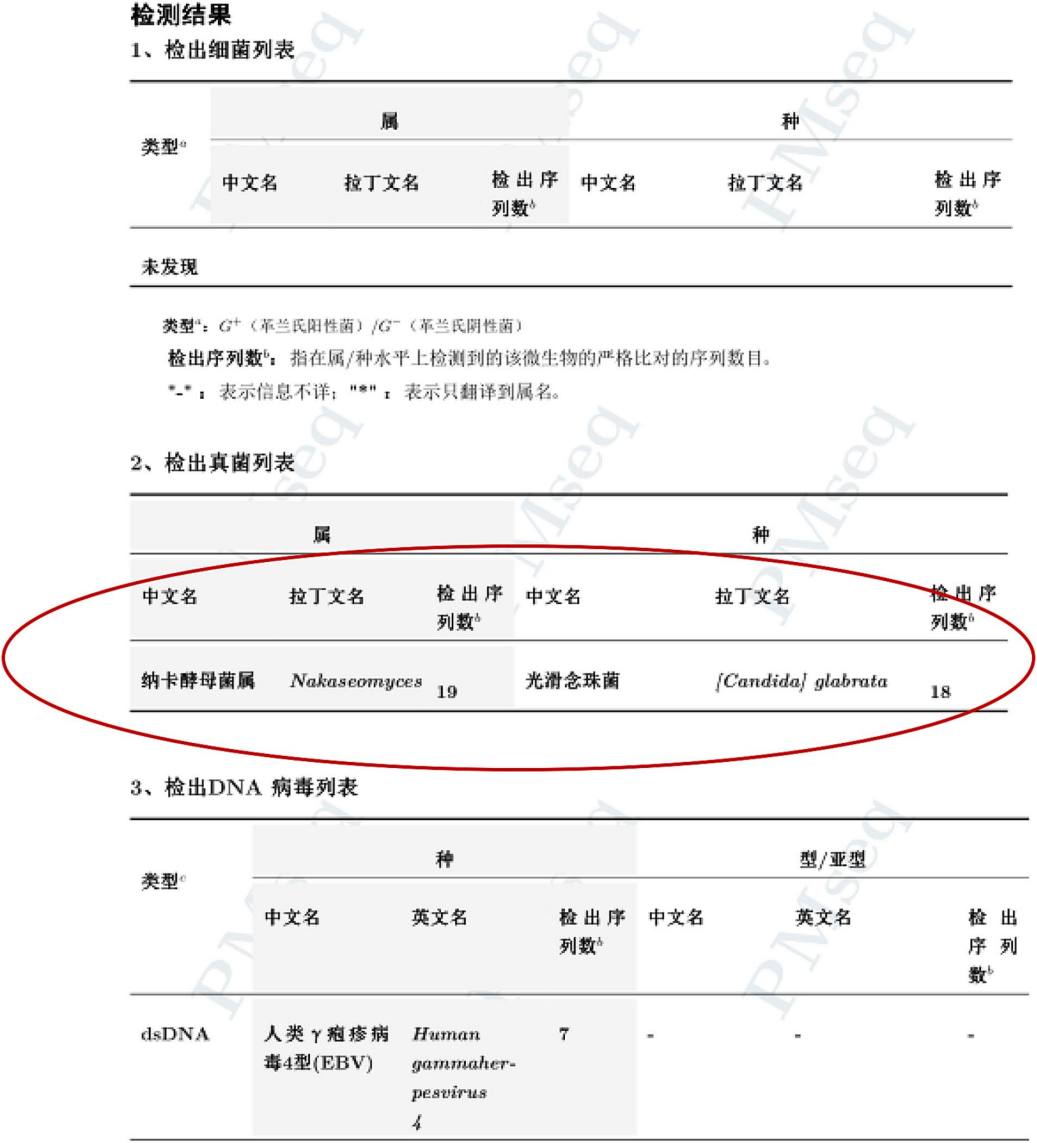
*Candida glabrata* infection confirmed by next-generation sequencing.

Postoperatively, we administered empirical anti-infective therapy pending culture results. The final culture result was *Candida glabrata*, a kind of fungus, and the medication regimen was adjusted accordingly (Figure 4). This conclusion was also confirmed by the results of Next-generation sequencing (NGS), performed on BGISEQ-100 platform (Figure 5). The mapping of the filter sequences was carried out using Burrows-Wheeler Alignment (Version: 0.7.10). After 2 weeks of anti-fungal treatment, the patient was discharged on day 24 after admission where the urine fungal culture results eventually came to normal.

In conclusion, the final diagnoses in our patient include EC secondary to a *Candida glabrata* UTI and peritonitis secondary to intraperitoneal (IP) bladder perforation caused by GC.

## 3 Discussion

### 3.1 Overview

Emphysematous cystitis is a serious UTI featured by an accumulation of gas in the bladder wall or bladder lumen, which results from fungal or bacterial fermentation (7). Although very little information regarding EC has been reported, with a mortality rate of 7%, EC acts as a rare but critical condition (8). In this scenario, not only did the patient have a 20-year history of DM with poor glycemic control, but she also had a recent indwelling urinary catheter, both of which were risk factors that contributed to her predisposition to EC.

### 3.2 Clinical manifestations and diagnosis

The clinical manifestations of EC vary widely and can range from asymptomatic to mild UTI symptoms or even to peritonitis and complete septic shock (9). Our patient first presented with acute abdominal pain and a sensation of snow-grip over the abdomen before septic shock appeared. The symptoms are vague and nonspecific, making the diagnosis extremely challenging, so radiographic imaging is required to confirm the diagnosis (2). Plain abdomen X-rays can show air fluid levels within the bladder on and/or a rim of gas lucency encircling the bladder wall. Nonetheless, CT scans are more effective in identifying cases that are not visible on conventional radiographs, assessing the severity and progress of the illness, and distinguishing EC from other illnesses, such as intra-abdominal abscesses and colovesical fistula (10). In this case, CT scans demonstrated a “beaded necklace appearance” along the bladder wall caused by an irregular thickening of the mucosal surface (Figure 2a). In addition to the bladder wall and lumen, hypointense shadows were visible at the upper and lower abdominal CT levels, such as under the chest and abdominal wall (Figures 2c,e), which could be the main contributor of the sensation to the snow-grip sensation in the abdomen. Despite the risk of rectal rupture was initially suspected (due to a suspected pelvic fracture), a negative DRE helped us to rule it out. Subsequent CT scans showing negative subdiaphragmatic free gas gave us the first impression of a retroperitoneal or interstitial organ perforation, further affirming the possibility of bladder perforation. As the surgery progressed, we ultimately confirmed the fact that gangrene and perforation of the bladder which was consistent with the images and symptoms.

### 3.3 Microbiology

The most common pathogen was discovered to be *E. coli* (60%), followed by *Klebsiella pneumonia* (10–20%). Simultaneously, *Clostridium perfringens*, *Candida*, and *Enterococcus* are additionally prevalent bacteria that can generate gas (11, 12). Fungus-induced EC cases are very uncommon, on the other hand, there have been almost no reported cases of EC caused by fungus leading to gangrene and perforation. In our case, EC resulting from a *Candida glabrata*-related UTI and generalized peritonitis secondary to an IP bladder perforation driven by GC comprised the patient’s ultimate diagnosis. The only other case of *Candida glabrata* causing EC and bladder rupture in a 68-year-old female with DM has been reported in *Urology Case Reports* by Sapkalova et al. on June 2023 (6). Unfortunately, the patient in that case eventually passed away due to respiratory and multiorgan failure. Similarly, the largest difference with another *Candida glabrata*-induced EC and GC is that we reported the first case of a successful management in this kind of patients, which could provide some guidance for the clinical treatment of these patients for the purpose of achieving a favorable prognosis.

### 3.4 Management

An empirical antibiotic treatment may be initially employed for emphysematous cystitis, and then antibiotic therapy may be adjusted according to the results of bacterial cultures. Additionally, urine drainage can assist the bladder reestablish its function and minimize inflammation as a crucial part of antibiotic-based therapy protocol (13). Nevertheless, EC can be potentially life-threatening when complicated by bladder perforation or septic shock, as in our case where the patient poorly responded to conservative treatment and developed a progressive drop in BP, in which case surgical intervention was required. Surgical managements with options determined by the severity of the disease range from cystectomy partial, cystectomy, surgical debridement or even nephrectomy in combined emphysematous pyelitis (EP) cases (1, 4). Overall, the prevalence of bladder rupture secondary to EC is quite low, with only 12 cases having been reported in the last decade, and we have also compiled them into a table to enable a convenient comparison of patient’s basic condition, management and prognosis in each case (Table 2). As we can note that the prevalence is slightly higher among females than males, but there does not seem to be a significant relationship with DM. Through appropriate treatment, the prognosis of these patients was fine except for one case of mortality. Furthermore, most of the causative organisms are still *E. coli*, followed by *Klebsiella pneumoniae*, which is consistent with the spectrum of pathogens in the EC. Therefore, antibiotic treatment is required in every patient, and catheterization also serves as an indispensable step in the management of EC, which can be seen in almost every case.

**Table 2:** Comparison of different recent cases of EC complicated with bladder rupture and their management published from 2012 to 2023.

Age	Sex	History of DM	Complications/ comorbidities	Microbiology	Management	Prognosis	References
60 years	M	Type 2 DM	Bilateral hydronephrosis and emphysematous pyelitis	*E. coli*	Antibiotic treatment, partial cystectomy, cystorrhaphy and catheterization	Survived (discharged on postoperative day 4)	Hudnall et al. (18) (2019)
55 years	F	None	Postoperative prolapsed intervertebral disc at L3–L4 region (history of urethral catheter)	*E. coli*	Antibiotic treatment, cystorrhaphy and catheterization	Survived (discharged after 8 days)	Velhal et al. (19) (2021)
84 years	M	None	Mesangial-proliferative glomerulonephritis (history of PD), BPH, hypertension and RA	*E. coli*	Antibiotic treatment, bladder irrigation, and catheterization	Survived (discharged after 10 days)	Okello et al. (20) (2021)
78 years	F	None	Parkinson’s Disease	*E. coli*	Antibiotic treatment, cystorrhaphy and catheterization	Survived (discharged on postoperative day 12)	Becker et al. (21) (2023)
64 years	M	None	BPH (history of a long-term urethral catheter) and hypertension	Negative	Empirical treatment and catheterization	Survived (discharged after 11 days)	Keeling et al. (22) (2021)
32 years	F	None	History of vacuum-assisted vaginal delivery before 5 days	*Enterococcus faecium* and *Acinetobacter baumannii*	Antibiotic treatment, bilateral cutaneous ureterostomy	Survived (discharged after 2 months)	Viswanathan et al. (23) (2012)
74 years	F	None	NR	NR	Antibiotic treatment and catheterization	Survived (spontaneous healing of the bladder wall after 1 week)	Roels et al. (24) (2016)
80 years	F	None	Terminal MS and intracerebral hemorrhage	*Klebsiella pneumoniae*	Antibiotic treatment and bladder irrigation	Survived (discharged after 22 days)	Kawahigashi et al. (25) (2023)
86 years	M	None	PAD and gangrene of the anterior abdominal wall	*Morganella morganii*	Antibiotic treatment and total cystectomy	Survived, but with a history of PE about 20 days postoperatively	Kouiss et al. (5) (2023)
70 years	F	Type 2 DM for 15 years	Generalized peritonitis	*E. coli*	Antibiotic treatment, cystorrhaphy and catheterization	Survived (discharged after 1 week)	Eghbali et al. (26) (2022)
80 years	F	Type 2 DM	Sepsis	*Klebsiella pneumoniae*	Antibiotic treatment, partial cystectomy and urine drainage	Survived (discharged after 2 months)	Zhang et al. (13) (2020)
68 years	F	Type 2 DM	PAD, CAD, AS	*Candida glabrata*	Antibiotic treatment, DKA protocol and catheterization	Died (after a 22-day stay)	Sapkalova et al. (6) (2023)

EC, emphysematous cystitis; DM, diabetes mellitus; M, male; F, female; *E. coli*, *Escherichia coli*; PD, peritoneal dialysis; BPH, benign prostate hypertrophy; RA, rheumatoid arthritis; NR, not recorded; MS, myelodysplastic syndrome; PAD, peripheral artery disease; PE, pulmonary embolism; CAD, coronary artery disease; AS, atherosclerosis; DKA, diabetic ketoacidosis.

### 3.5 Pathogenesis

#### 3.5.1 Exploration into occurrence of gas-forming infection in urinary tract

**Figure 6:**
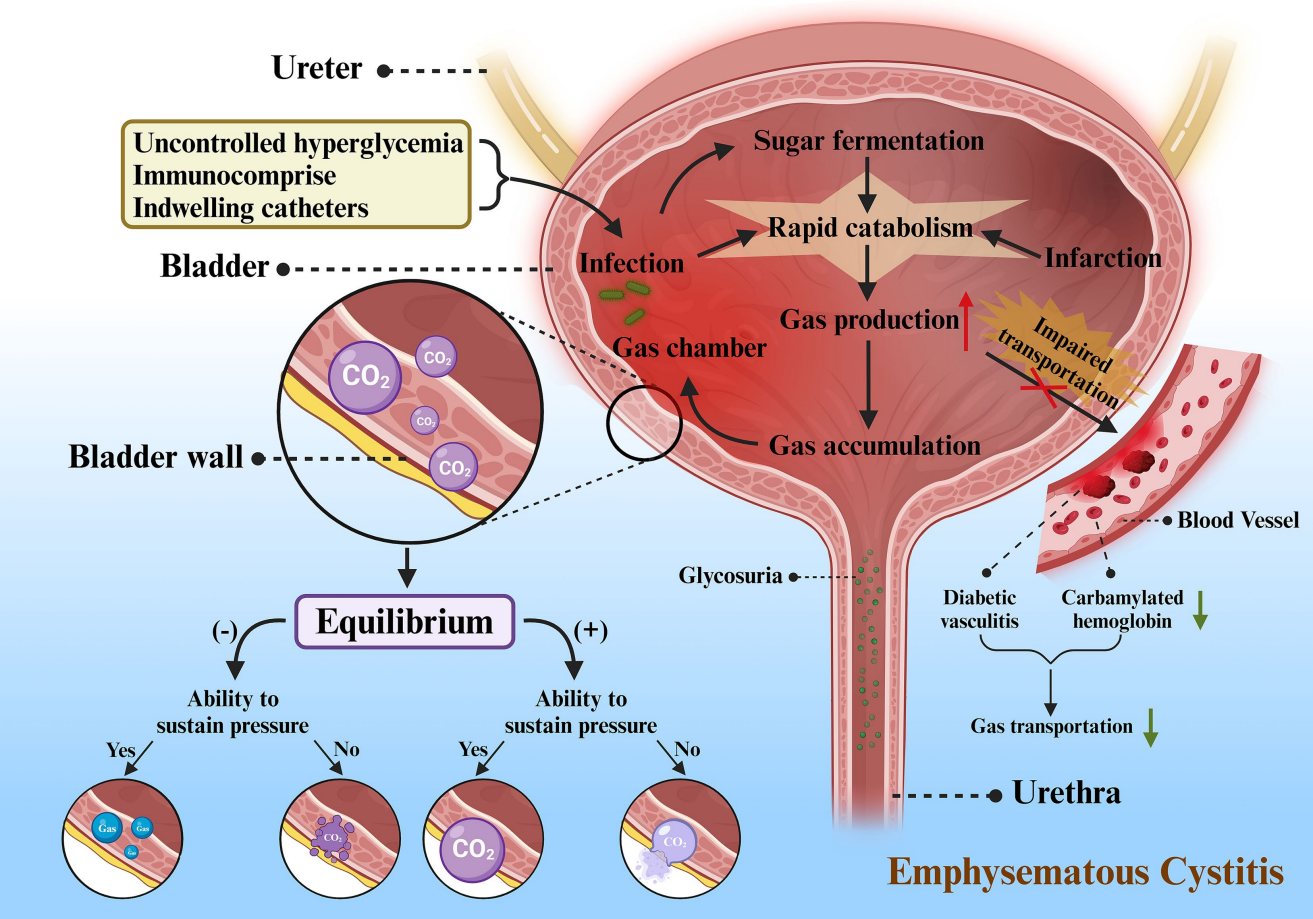
Proposed mechanism for emphysematous cystitis. Firstly, uncontrolled hyperglycemia, immunocompromise, and indwelling catheters serve as important incentives for infection. Sugar fermentation and the microorganisms can accelerate catabolism after the onset of infection. Tissue infarction induced by infection also leads to rapid catabolism, resulting in increased gas production. As a result of poor circulation (diabetic vasculitis) and impaired gas transport (decreased carbamylated hemoglobin), there is a decline in gas transportation and an accumulation of gas, which ultimately creates gas chambers in the bladder wall, most of which are CO_2_. The gas in the chamber follows the laws of equilibrium for different outcomes: **(1)** When negative equilibrium occurs and the gas chamber is able to withstand the pressure, CO_2_ will be replaced by gas from adjacent tissues, but the chamber persists. **(2)** When negative equilibrium occurs and the gas chamber is unable to withstand the pressure, gas chamber will collapse and finally disappear. **(3)** When positive equilibrium occurs and the gas chamber is able to withstand the pressure, gas chamber will gradually enlarge. **(4)** When positive equilibrium occurs and the gas chamber is unable to withstand the pressure, gas chamber will eventually rupture into neighboring tissues or cavum abdominis.

Apparently uncontrolled hyperglycemia, immunocompromise and indwelling catheters contributed to the opportunistic infection of *Candida glabrata* in our patient’s bladder. However, where did the gas come from? Currently, the exact mechanism of gas-forming infection of the urinary tract remains still not unclear hence numerous theories exist for its explanation. Yet, there was a hypothesis proposed by Yang et al., which we could refer to as the “Gas Equilibrium Hypothesis,” that was known to better illustrate the pathogenesis of EC (14). In this theory, it incorporates increased gas production, compromised gas transport, generation of gas chambers, equilibrium between tissue gases and the gases in the chambers, and collapse of the chambers as the principal components of the pathogenesis. Rapid catabolism and impaired transportation serve as the two predominant factors for increased gas production and accumulation (Figure 6).

#### 3.5.2 Rapid catabolism

There are various factors triggering rapid catabolism, such as severe infections, tissue infarcts, or sugar fermentation. This gas can be produced from nitrogen, hydrogen, oxygen and carbon dioxide (CO_2_), but usually CO_2_ (15). As glucose constitutes one of the main substrates for natural fermentation by microbes, diabetes may play a major role in increasing the formation of CO_2_. While diabetes reduces the host’s immunity, it also leads to high glucose levels in tissues, urine, and cystic fluid, which creates a favorable incubation environment for infectious microorganisms. In addition, infarction induces rapid catabolism, together with the impaired circulation inherently leading to the massive generation of CO_2_ and accumulation of gases in the tissues.

#### 3.5.3 Impaired transportation

Impaired transportation can present in two forms, impaired transportation of gases in the blood and poor circulation. As an example, our patients might be prone to poor circulation as a result of diabetic vasculitis. In addition, on account of the fact that a small portion of CO_2_ exists in the blood either in gaseous form or bound to hemoglobin, i.e., carbamylated hemoglobin (CHB), hyperglycemia in diabetic patients with poor glycemic control may potentially result in the glycosylation of carbonic anhydrase, which can further worsen the circulation through decreasing enzyme activity (16).

Usually there are two chief factors that lead to these two manifestations, respectively, infection and obstruction. Severe infections can elevate the pressure in the bladder lumen, impeding circulation and diminishing gas delivery. The increased secretions and exudates produced by the infection can act as a hindrance to gas transportation, thereby raising local pressures and further jeopardizing the already poor circulation. Accumulation of gas will further contribute to localized pressure and may even entail infarction of neighboring tissues, which serves as an obstacle not only to gas transport, but also to the supply of a good medium, as well as to the enhanced catabolism that can yield even more gas. On the other hand, urinary tract obstruction not only causes urine stasis, contributing to severe infections and rapid catabolism, but also increases localized pressure in the bladder lumen and compromises circulation. Both of these become a vicious cycle that will continue and may even be fatal if the whole process is not interrupted.

#### 3.5.4 Gas equilibrium between the chamber and local tissue

As the pressure increases, the buildup of gas can expand the localized tissue and create a bubble-forming chamber within which the gas is initially a product of the infection. From a physiological point of view, every reaction in the body tends to reach equilibrium. Thus, the gases in the tissues or surrounding material will come into equilibrium with the gases generated by the infection within the chamber over time. From a physiological point of view, every reaction in the body tends to reach equilibrium. Thus, the gases in the tissues or peripheral substances will come into equilibrium with the gases generated by the infection within the chamber over time. Eventually, the products of equilibrium between the gas and the local tissues at this time may constitute the composition of the gas within the chamber.

When positive equilibrium happens, the chamber will continue to expand. Nonetheless, if it dilates beyond the chamber’s capacity, it may rupture into neighboring tissues and form a fresh chamber. Sometimes, it can also break into the cavum abdominis, with bladder rupture occurring as in this case. Since the wall of the chamber can be a portion of the cystic wall, a partial bladder wall or sometimes necrotic tissues, EC sometimes merge with GC (5), which can also be reflected in our case.

Negative equilibrium occurs as the absorption of gas exceeds the production and intake of gas, and it plays a pivotal step in the elimination of the chamber. Another key refers to chamber collapse, which occurs when the pressure inside the chamber drops below the outside pressure and the strength of the chamber cannot sustain it, the chamber will collapse with the possibility of disappearance. Exceptionally, there are cases in which the gas chamber is robust enough to withstand pressure from the outside and the bubble does not disappear even if all the gas is replaced by tissue-derived gas. This explanation also accounts for the persistence of gas in some cases without additional signs of toxicity after treatment (17).

## 4 Conclusion

EC is a rare disease that mainly affects patients with uncontrolled diabetes, especially elderly females, but it can be life-threatening when complicated with gangrene and bladder perforation. We are not only the first to report a case of EC combined with perforation secondary to *Candida glabrata* infection and successfully saved, but also the first to discuss the underlying mechanisms of EC pathogenesis. Nevertheless, these findings were from only one patient, which is insufficient to provide detailed instructions for the clinical management of EC combined with GC and perforation. Further studies on more patients and the mechanism of EC development are still necessary.

In general, owing to the high risk of bladder perforation in EC, we consider that the description of this case and its approach to enable other clinicians to have a higher index of suspicion when evaluating at-risk patients with similar symptoms.

## Data Availability

The original contributions presented in the study are included in the article/supplementary material, further inquiries can be directed to the corresponding authors.
